# Novel Bat Alphacoronaviruses in Southern China Support Chinese Horseshoe Bats as an Important Reservoir for Potential Novel Coronaviruses

**DOI:** 10.3390/v11050423

**Published:** 2019-05-07

**Authors:** Susanna K.P. Lau, Antonio C.P. Wong, Libao Zhang, Hayes K.H. Luk, Jamie S. L. Kwok, Syed S. Ahmed, Jian-Piao Cai, Pyrear S.H. Zhao, Jade L.L. Teng, Stephen K.W. Tsui, Kwok-Yung Yuen, Patrick C. Y. Woo

**Affiliations:** 1 Department of Microbiology, Li Ka Shing Faculty of Medicine, The University of Hong Kong, Pokfulam, Hong Kong, China; skplau@hku.hk (S.K.P.L.); antonwcp@connect.hku.hk (A.C.P.W); hkhluk@hku.hk (H.K.H.L.); shakeel87@gmail.com (S.S.A.); caijuice@hku.hk (J.-P.C.); pyrear@126.com (P.S.H.Z.); llteng@hku.hk (J.L.L.T.); 2 State Key Laboratory of Emerging Infectious Diseases, The University of Hong Kong, Pokfulam, Hong Kong, China; 3 Carol Yu Centre for Infection, The University of Hong Kong, Pokfulam, Hong Kong, China; 4 Collaborative Innovation Centre for Diagnosis and Treatment of Infectious Diseases, The University of Hong Kong, Pokfulam, Hong Kong, China; 5 Guangdong Key Laboratory of Animal Conservation and Resource Utilization, Guangdong Public Laboratory of Wild Animal Conservation and Utilization, Guangdong Institute of Applied Biological Resources, Guangzhou 510000, China; zhanglb@giabr.gd.cn; 6 School of Biomedical Sciences, Faculty of Medicine, The Chinese University of Hong Kong, Shatin, Hong Kong, China; jamie_slk@link.cuhk.edu.hk (J.S.L.K.); kwtsui@cuhk.edu.hk (S.K.W.T.)

**Keywords:** *Alphacoronavirus*, novel, bat, coronavirus, species, molecular, discovery, epidemics

## Abstract

While bats are increasingly recognized as a source of coronavirus epidemics, the diversity and emergence potential of bat coronaviruses remains to be fully understood. Among 1779 bat samples collected in China, diverse coronaviruses were detected in 32 samples from five different bat species by RT-PCR. Two novel alphacoronaviruses, *Rhinolophus sinicus* bat coronavirus HKU32 (Rs-BatCoV HKU32) and *Tylonycteris robustula* bat coronavirus HKU33 (Tr-BatCoV HKU33), were discovered from Chinese horseshoe bats in Hong Kong and greater bamboo bats in Guizhou Province, respectively. Genome analyses showed that Rs-BatCoV HKU32 is closely related to BatCoV HKU10 and related viruses from diverse bat families, whereas Tr-BatCoV HKU33 is closely related to BtNv-AlphaCoV and similar viruses exclusively from bats of *Vespertilionidae* family. The close relatedness of Rs-BatCoV HKU32 to BatCoV HKU10 which was also detected in Pomona roundleaf bats from the same country park suggests that these viruses may have the tendency of infecting genetically distant bat populations of close geographical proximity with subsequent genetic divergence. Moreover, the presence of SARSr-CoV ORF7a-like protein in Rs-BatCoV HKU32 suggests a common evolutionary origin of this accessory protein with SARS-CoV, also from Chinese horseshoe bats, an apparent reservoir for coronavirus epidemics. The emergence potential of Rs-BatCoV HKU32 should be explored.

## 1. Introduction

The Severe Acute Respiratory Syndrome (SARS) and more recently the Middle East Respiratory Syndrome (MERS) have proven the emergence potential of animal coronaviruses (CoVs) and aroused immense interest in the discovery of novel CoVs in animals and humans. While SARS coronavirus (SARS-CoV) was originated from horseshoe bats in China as its animal reservoir and transmitted to humans after amplification in palm civets from wildlife markets [1,2], dromedary camels in the Middle East are the immediate animal source of the MERS epidemic caused by MERS coronavirus (MERS-CoV) [3,4,5,6]. Bats also harbor MERS-CoV-related viruses, which may suggest a possible bat origin [7,8,9,10,11,12,13,14,15], although the evolutionary origin of MERS-CoV remains to be ascertained.

Through the discovery of numerous novel CoVs since the SARS epidemic [16,17,18], bats were uncovered as an important animal reservoir for alphacoronaviruses (alphaCoVs) and betacoronaviruses (betaCoVs), and birds as an important reservoir for gammacoronaviruses (gammaCoVs) and deltacoronaviruses (deltaCoVs) [19,20,21,22,23]. In particular, bats harbor CoVs that can evolve to cause epidemics in humans and other animals. When MERS-CoV was first discovered, it was most closely related to *Tylonycteris* bat CoV HKU4 (Ty-BatCoV HKU4) and *Pipistrellus* bat CoV HKU5 (Pi-BatCoV HKU5) that were detected five years ahead of the MERS epidemic, from bats in Hong Kong [7,8,9,10,24,25]. This illustrates the importance of continuous surveillance studies of bat CoVs in preparing for future epidemics in humans.

Besides SARS-CoV and MERS-CoV, bat CoVs closely related to other human CoVs, including human CoV 229E and human CoV NL63, were also recently discovered [26,27,28], suggesting that bats are the important animal source of CoVs that may emerge in humans. On the other hand, bat CoVs may also evolve to infect other animals. For example, porcine epidemic diarrhea virus (PEDV) is phylogenetically closely related to *Scotophilus* bat coronavirus 512 (Sc-BatCoV 512), suggesting cross-species transmission events between bats and pigs [29]. In 2016–2017, outbreaks of severe watery diarrhea were reported in suckling piglets from farms in Guangdong Province, China, which were found to be caused by swine acute diarrhea syndrome coronavirus (SADS-CoV) [30,31,32]. SADS-CoV is very close to and likely to have emerged from *Rhinolophus* bat CoV HKU2 (Rh-BatCoV HKU2), first discovered in Hong Kong and detected in a wide range of horseshoe bats including *Rhinolophus sinicus*, *Rhinolophus affinis* and *Rhinolophus ferrumequinum* [30,33]. In particular, the spike protein of SADS-CoV shared 93–98% amino acid identity to that of Rh-BatCoV HKU2 from *Rhinolophus affinis*, supporting recent interspecies jumping from bats to pigs [30].

To further explore the diversity of CoVs in bats and understand the genetic evolution of CoVs, we collected bat samples from Hong Kong and mainland China. Diverse CoVs belonging to alphaCoVs and betaCoVs were detected, including two novel alphaCoVs, as confirmed by complete genome sequencing and characterization, supporting bats as an important reservoir for CoVs. The evolutionary relationship of the two novel alphaCoVs to other known CoVs is also discussed.

## 2. Materials and Methods

### 2.1. Ethics Statement

Collection of bat samples in Hong Kong was approved by the Department of Agriculture, Fisheries and Conservation, Hong Kong Special Administrative Region (HKSAR); and the Committee on the Use of Live Animals in Teaching and Research, The University of Hong Kong (CULATR Ref. No.: 2284-10 and 3330-14; Date of approval: 23 March 2011 and 17 April 2014). Bat samples from mainland China were collected by the Guangdong Institute of Applied Biological Resources (Guangzhou, China) in accordance with guidelines of Regulations for Administration of Laboratory Animals under a license from Guangdong Entomological Institute Administrative Panel on Laboratory Animal Care.

### 2.2. Collection of Bat Samples

Oral and alimentary samples were collected from bats captured from HKSAR, and Guizhou and Guangdong Provinces, mainland China, during 2013−2015 using procedures described previously [1]. To prevent cross contamination, sterile disposable swabs with protective gloves were used during sample collection and changed between samples. All samples were immediately placed in viral transport medium (Earle’s balanced salt solution, 20% glucose, 4.4% NaHCO_3_, 5% bovine albumin, vancomycin 50,000 µg/mL, amikacin 50,000 µg/mL, nystatin 10,000 units/mL) before transportation to the laboratory. For prolonged storage, all samples were stored at −80 °C before further studies.

### 2.3. Detection of Bat CoVs by RNA Extraction, RT-PCR and DNA Sequencing

Viral RNA was extracted form oral and alimentary samples using QIAamp viral RNA minikit (Qiagen, Hilden, Germany). Eluted RNA was used as the template for reverse transcription-PCR (RT-PCR). Detection of CoV was performed by amplifying a 440-bp fragment of the RNA-dependent RNA polymerase (RdRp) gene of CoVs using conserved primers (5’-GGTTGGGACTATCCTAAGTGTGA-3’ and 5’-ACCATCATCNGANARDATCATNA-3’) [18]. Reverse transcription was performed using a SuperScript III kit (Invitrogen, San Diego, CA, USA). PCR mixture (25 µL) was prepared and PCR conditions were set as described previously in an automated thermal cycler (Applied Biosystems) [34]. Amplified PCR products were gel-purified using the QIAquick gel extraction kit (QIAgen). Both strands of PCR products were sequenced with an ABI Prism 3130x genetic Analyzer (Applied Biosystems, Foster City, CA, USA), using the above primers. Comparison of the PCR products’ sequences with other known CoVs’ RdRp genes from GenBank sequence database was performed.

### 2.4. Viral Culture

Attempts to isolate Rs-BatCoV HKU32 and Tr-BatCoV HKU33 were performed by inoculating samples with RT-PCR positive results to different cells. Viral replication was detected by cytopathic effect observation and viral detection of culture supernatant collected from passages by RT-PCR.

### 2.5. Complete Genome Sequencing of Rs-BatCoV HKU32 and Tr-BatCoV HKU33

Viral genomes of two Rs-BatCoV HKU32 (TLC26A and TLC28A) and one Tr-BatCoV HKU33 (GZ151867) were amplified and sequenced using RNA directly extracted from their alimentary samples respectively as templates. Both viral RNAs were reverse transcribed to cDNA by a combined random-priming and oligo(dT)-priming strategy. For GZ151867, the amplified cDNA sample was barcoded and sequenced using the Ion Torrent sequencing platform. The average sequencing throughput of these samples was 12.90 Mbp and the average read length was 150.1 bp. The single-end reads were de novo assembled using SPAdes Genome Assembler version 3.10.0 using default parameters [35]. Coronavirus-matching contigs were searched using BLASTN version 2.5.0+ against NCBI nucleotide database (nt) version downloaded on October 4th 2016 with e-value cutoff at 1 × 10^−5^ [36].

Degenerated primers were designed according to multiple alignments of the genomes of other alphaCoVs with complete genomes available, using strategies as described previously [18,20,37]. Additional primers were designed based on the results of the first and subsequent rounds of sequencing or the NGS sequencing for the sample GZ151867. SMARTer 5’/3’ RACE kit (Clontech, Mountain View, CA, USA) was used to perform rapid amplification of cDNA ends and confirm the 5’ ends of the genomes. For Rs-BatCoV HKU32, a total of 53 sets of primers (available on request) were used for PCR. For Tr-BatCoV HKU33, a total of 44 sets of primers (available on request) were used for PCR. Sequences were assembled and edited manually to produce complete sequences of the three viral genomes.

### 2.6. Phylogenetic and Genome Analysis of Rs-BatCoV HKU32 and Tr-BatCoV HKU33

The genomes of Rs-BatCoV HKU32 and Tr-BatCoV HKU33 were aligned and analyzed with other alphaCoVs with complete genome sequences available from Genbank using online sequence alignment server MAFFT version 7 [38]. The nucleotide sequences of the genomes and the deduced amino acid sequences of the open reading frames were analyzed and compared with other alphaCoVs using ORFfinder (https://www.ncbi.nlm.nih.gov/orffinder/). Maximum-likelihood phylogenetic tress with 1000 bootstrap replicates of ORF1ab and S genes were constructed using PhyMLv3.0 (The French Institute of Bioinformatics & France Genomique, Montpellier, France) [39]. Smart Model Selection from PhyML was used to calculate the best-fit substitution model for ML analyses.

### 2.7. Expression of ORF10 Accessory Gene and Determination of Leader-Body Junction Sequence

The leader-body junction site and flanking sequences of the ORF10 subgenomic mRNA in Rs-BatCoV HKU32 strain TLC28A was sequenced and determined using RT-PCR method as described previously. cDNA obtained from RT was used as the template for PCR amplification with a forward primer (5’-GCGTCTCATCCCCTCAA-3’) located in the leader sequence and a reverse primer (5’-GAACCAGCGATACAATCAATG-3’) located in the body of the ORF10 subgenomic mRNA. PCR mixture was prepared as described previously. The mixtures were amplified for 60 cycles of 94 °C for 1 min, 55 °C for 1 min, and 72 °C for 1 min and a final extension at 72 °C for 10 min. Amplified PCR products were subjected to gel purification and sequencing as described previously.

### 2.8. Accession Number

The nucleotide sequences of the three complete genomes of Rs-BatCoV HKU32 and Tr-BatCoV HKU33 have been deposited in the GenBank sequence databases with the accession numbers MK720944 to MK720946.

## 3. Results

### 3.1. Bat Coronaviruses Surveillance and Identification of Two Novel Alphacoronaviruses

**Figure 1:**
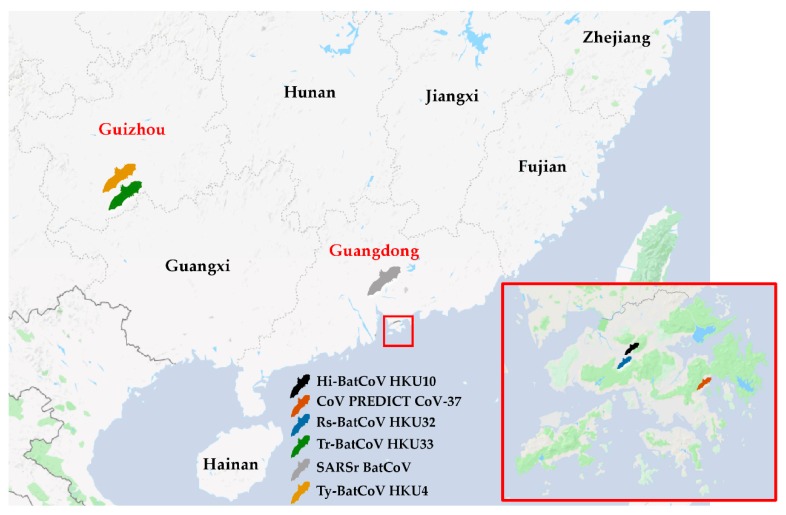
Map of southern China showing locations where bat coronaviruses were found. Black bat represents the location with bats positive for Hi-BatCoV HKU10; orange bat represents the location with bats positive for coronavirus (CoV) PREDICT CoV-37; blue bat represents the location with bats positive for Rs-BatCoV HKU32; green bat represents the location with bats positive for Tr-BatCoV HKU33; grey bat represents the location with bats positive for severe acute respiratory syndrome related (SARSr) BatCoV; yellow bat represents the location with bats positive for Ty-BatCoV HKU4. Provinces where samples were collected are in red font.

A total of 1779 alimentary samples from 1117 bats of 20 species were obtained from Hong Kong and Guangdong and Guizhou Provinces in southern China (Figure 1, Table 1). RT-PCR for a 440-bp fragment of RdRp gene of CoVs was positive in samples from 32 bats (2.9%) of 5 species belonging to 4 genera. Sequence analysis showed that 11 samples contained alphaCoVs, 3 contained *Sarbecovirus* (lineage B betaCoVs) and 18 contained *Merbecovirus* (lineage C betaCoVs).

7 alphaCoV sequences from *Rhinolophus sinicus* (Chinese horseshoe bats) captured in Hong Kong showed ≤84% nt identity to the corresponding sequences of BtRfAlphaCoV/HuB2013 (GenBank accession no. NC_028814.1) and other alphaCoVs, suggesting a potentially novel alphaCoV proposed to be named *Rhinolophus sinicus* bat coronavirus HKU32 (Rs-BatCoV HKU32) (Table 1). One other alphaCoV sequence from *Tylonycteris robustula* (Greater bamboo bats) captured from Luodian County in Guizhou Province showed ≤81% nt identity to the corresponding sequence of BtNv-AlphaCoV/SC2013 (GenBank accession no. NC_028833.1) and other alphaCoVs, suggesting another potentially novel alphaCoV proposed to be named *Tylonycteris robustula* bat coronavirus HKU33 (Tr-BatCoV HKU33) (Table 1). Attempts to isolate both Rs-BatCoV HKU32 and Tr-BatCoV HKU33 in Vero, VeroE6, RSK (in-house development), RSL (in-house development), HeLa, Caco-2 and HT-29 cells were unsuccessful. No cytopathic effect or viral replication was detected.

The other positive bat samples contained known bat alphaCoVs and betaCoVs. Hi-BatCoV HKU10 was detected in 2 samples from *Hipposideros pomona* captured in Hong Kong, with 99% nucleotide identity to the corresponding partial RdRp sequence of Hi-BatCoV HKU10 isolate TLC1310A (GenBank accession no. JQ989268.1) (Table 1). *Myotis daubentonii* CoV was detected in 1 sample from *Myotis ricketti* captured in Hong Kong, sharing 96% nucleotide identity to Coronavirus PREDICT CoV-37 (GenBank accession no. KX285138.1) (Table 1). For betaCoVs, 3 samples from *Rhinolophus sinicus* captured in Guangdong Province contained SARS-related BatCoVs (*Sarbecovirus*) with 99% nucleotide identity to SARS-related BatCoV HKU3-12 (GenBank accession no. GQ153547.1) (Table 1). Ty-BatCoV HKU4 *(Merbecovirus)* was detected in 18 samples of *Tylonycteris pachypus* captured in Guizhou Province, with 95–96% nucleotide identity to Ty-BatCoV HKU4-4 (GenBank accession no. EF065508.1) (Table 1).

### 3.2. Genome Features of the Two Novel Alphacoronaviruses, Rs-BatCoV HKU32 and Tr-BatCoV HKU33

The complete genomes of two strains of Rs-BatCoV HKU32, TLC26A and TLC28A, and Tr-BatCoV HKU33 strain GZ151867 were sequenced and determined to characterize their genome features.

#### 3.2.1. Novel alphaCoV Species: Rs-BatCoV HKU32

Both genomes of Rs-BatCoV HKU32 TLC26A and TLC28A possessed genome sizes of 29201 nucleotides, with 40.3% G + C content. They shared 99% overall nucleotide identity to each another. Rs-BatCoV HKU32 strain TLC28A was selected as the reference strain for the following genomic analyses.

**Figure 2:**
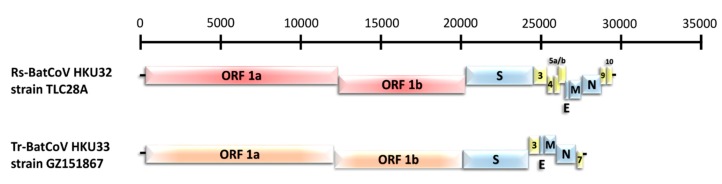
Genome organizations of Rs-BatCoV HKU32 strain TLC28A and Tr-BatCoV HKU33 strain GZ151867. Genes for ORF 1a and 1b of Rs-BatCoV HKU32 strain TLC28A and Tr-BatCoV HKU33 strain GZ151867 are represented by red and orange boxes, respectively. Genes for spike protein (S), envelope protein (E), membrane protein (M) and nucleocapsid protein (N) are represented by blue boxes. Genes for putative accessory proteins are represented by yellow boxes.

Similar to other alphaCoV genomes, Rs-BatCoV HKU32 consisted of 10 putative open reading frames (ORFs) including the essential ORF1ab, S, E, M and N (Table 2). 3 accessory genes were located between the S and N genes while 2 accessory genes were found downstream of N gene (Figure 2). Rs-BatCoV HKU32 ORF3 accessory protein shared low amino acid identity (31%–51%) to the respective accessory proteins in other alphaCoVs while Rs-BatCoV HKU32 ORF10 accessory protein shared 29% amino acid identity to SARSr-CoV ORF7a accessory protein. A putative transcription regulatory sequence (TRS) motif, 5’-CUAAAC-3’, was identified at the 3’ end of the leader sequence and preceded most ORFs except the S, ORF3, ORF5a and ORF9 (Table 2). An alternative TRS motif for S and ORF5 genes was found to be 5’-CUAAAU-3’, while that for ORF3 and ORF9 was 5’-CUAAAU-3’ and 5’-CUGAAC-3’, respectively (Table 2). The characteristics of putative nonstructural protein and predicted putative cleavage sites of Rs-BatCoV HKU32 are shown in Table 3 and Table 4.

Comparative genomic analyses showed that Rs-BatCoV HKU32 shared 58.9% overall nucleotide identity with BtRf-AlphaCoV and 67.2% with Hi-BatCoV HKU10. To determine whether Rs-BatCoV HKU32 was a novel alphaCoV species, 7 conserved replicase domains of Rs-BatCoV HKU32 were selected for analyses according to the CoV species demarcation criteria by the ICTV [23]. Five known alphaCoVs with complete genome sequences available and close phylogenetic relationship to Rs-BatCoV HKU32 were chosen for comparison. The 7 concatenated domains of Rs-BatCoV HKU32 shared 83.2%, 83.3%, 78.6% 70.3 and 69.5% amino acid identity with those of BtRf-AlphaCoV, BtMs-AlphaCoV, Ro-BatCoV HKU10, PEDV and Tr-BatCoV HKU33, respectively, which were below the threshold of 90% amino acid identity (Table 5). The results supported that Rs-BatCoV HKU32 represents a novel CoV species in the AlphaCoV genus.

#### 3.2.2. Novel alphaCoV Species: Tr-BatCoV HKU33

Tr-BatCoV HKU33 strain GZ151867 possessed a smaller genome size of 27,636 nucleotides compared to Rs-BatCoV HKU32 with 37% G + C content. It possessed 7 putative ORFs with only 2 accessory genes, ORF3 and ORF7 (Figure 2). The putative TRS motif of Tr-BatCoV HKU33 was 5’-CUAAAC-3’ and preceded ORF1ab, E, M and N. An alternative TRS motif for S and ORF7 genes was found to be 5’-CUAAAU-3’ while the alternative TRS motif for ORF3 and E was 5’-CUCAAC-3’ (Table 6). The characteristics of putative nonstructural proteins and predicted putative cleavage sites of Tr-BatCoV HKU33 were observed (Table 3 and Table 4).

Comparative genomic analyses showed that Tr-BatCoV HKU33 had the highest genome similarity with BtNv-AlphaCoV, sharing 69.4% overall nucleotide identity. Pairwise comparison of the 7 conserved replicase domains of Tr-BatCoV HKU33 indicated that Tr-BatCoV HKU33 shared 76.3% and 74.4% amino acid identity with its closest relatives BtNv-AlphaCoV and AlphaCoV BatCoV/P.kuhlii/Italy206679-3/2010, respectively, suggesting Tr-BatCoV HKU33 represents another novel CoV species in the AlphaCoV genus (Table 7).

### 3.3. Phylogenetic Analyses

**Figure 3:**
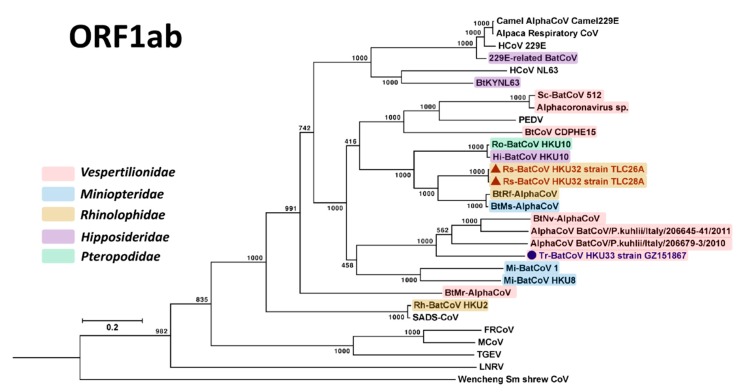
Phylogenetic analysis of ORF1ab amino acid sequences of Rs-BatCoV HKU32 strains TLC26A and 28A, Tr-BatCoV HKU33 strain GZ151867 and other alphaCoVs. ORF1ab tree was constructed by maximum likelihood method using LG + G + I + F substitution model. The bootstrap values are calculated from 1000 trees. Tree was rooted using corresponding sequence of Middle East respiratory syndrome (MERS)-CoV (GenBank accession number YP_009047202.1). All bootstrap values are shown. The scale bar represents 5 substitutions per site. Both Rs-BatCoV HKU32 and Tr-BatCoV HKU33 are labeled with red (triangle) and blue (circle), respectively. Corresponding viral bat hosts’ families are highlighted in different colors: Red, *Vespertilionidae*; blue, *Miniopteridae*; yellow, *Rhinolophidae*; purple, *Hipposideridae*; green, *Pteropodidae*.

**Figure 4:**
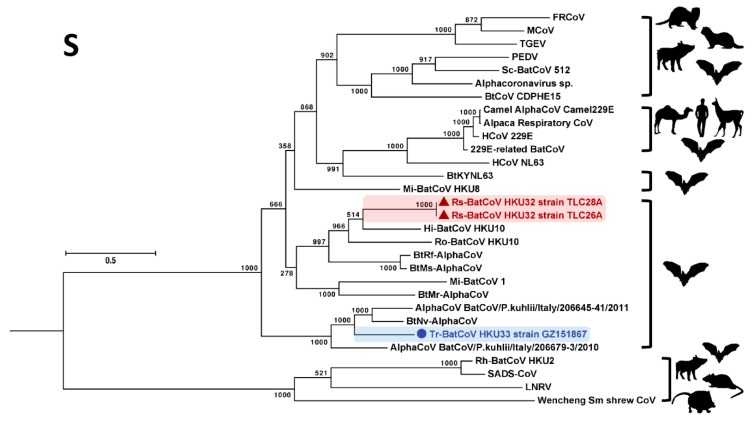
Phylogenetic analysis of S amino acid sequences of Rs-BatCoV HKU32 strains TLC26A and 28A, Tr-BatCoV HKU33 strain GZ151867 and other alphaCoVs. S tree was constructed by maximum likelihood method using WAG + G + I + F substitution model. The bootstrap values are calculated from 1000 trees. Tree was rooted using corresponding sequence of MERS-CoV (GenBank accession number YP_009047204.1). All bootstrap values are shown. The scale bar represents 2 substitutions per site. Both Rs-BatCoV HKU32 and Tr-BatCoV HKU33 are highlighted in red (triangle) and blue (circle), respectively. Corresponding viral hosts are shown on the right.

Phylogenetic trees were constructed using the amino acid sequences of ORF1ab and S proteins of Rs-BatCoV HKU32, Tr-BatCoV HKU33 and other alphaCoVs as shown in Figure 3 and Figure 4. For ORF1ab, Rs-BatCoV HKU32 formed a cluster with BtRf-AlphaCoV, BtMs-AlphaCoV, Ro-BatCoV HKU10 and Hi-BatCoV HKU10, being more closely related to BtRf-AlphaCoV and BtMs-AlphaCoV than to Ro-BatCoV HKU10 and Hi-BatCoV HKU10. Tr-BatCoV HKU33 was most closely related to and formed another cluster with BtNv-AlphaCoV, AlphaCoV BatCoV/P.kuhlii/Italy/206645-41/2011 and AlphaCoV BatCoV/P.kuhlii/Italy/206679-3/2010, but as an outlier branch at the root of this cluster (Figure 3).

In the S gene, Rs-BatCoV HKU32 and Tr-BatCoV HKU33 formed two similar clusters with related alphaCoVs but showed a different phylogenetic positioning compared to that in ORF1ab (Figure 4). Phylogenetically, Rs-BatCoV HKU32 was more closely related to Hi-BatCoV HKU10, and Ro-BatCoV HKU10 than to BtRf-AlphaCoV and BtMs-AlphaCoV. On the other hand, Tr-BatCoV HKU33 formed an inner branch within the cluster with BtNv-AlphaCoV, AlphaCoV BatCoV/P.kuhlii/Italy/206645-41/2011 and AlphaCoV BatCoV/P.kuhlii/Italy/206679-3/2010.

It is interesting to note that the AlphaCoV cluster formed by Rs-BatCoV HKU32, BatCoV HKU10, BtRs-AlphaCoV and BtMs-AlphaCoV was detected from diverse bat hosts from different bat families including *Pteropodidae*, *Hipposideridae*, *Rhinolophidae* and *Miniopteridae*. In contrast, the other cluster formed by Tr-BatCoV HKU33, BtNv-AlphaCoV, AlphaCoV BatCoV/P.kuhlii/Italy/206645-41/2011 and AlphaCoV BatCoV/P.kuhlii/Italy/206679-3/2010 detected from bats belonging to a single bat family, *Vespertilionidae* (Figure 3).

### 3.4. Homologous SARSr-CoV ORF7a-Like Accessory Protein in Rs-BatCoV HKU32

A homologous SARSr-CoV ORF7a-like accessory protein, ORF10, was found in Rs-BatCoV HKU32, located at nucleotide position 28593 to 28955 with 120 amino acids. The ORF7a accessory gene (also known as X4) found in SARSr-CoVs from both human and animal sources is a type I transmembrane protein [40]. InterProScan analysis showed that the Rs-BatCoV HKU32 ORF10 protein also possessed four domains, an N-terminal signal peptide, followed by a luminal domain, transmembrane segment and cytoplasmic tail. Rs-BatCoV HKU32 ORF10 protein possessed 29% amino acid identity to SARSr-CoV ORF7a protein.

**Figure 5:**
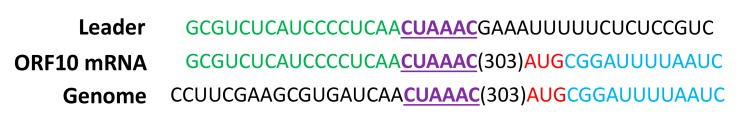
Rs-BatCoV HKU32 strain TLC28A mRNA leader-body junction and flanking sequences. The subgenomic ORF10 mRNA sequence are shown in alignment with the leader and genomic sequences. Identical nucleotides between the leader sequence and subgenomic mRNA sequence are labeled in green. Identical nucleotides between genome and subgenomic mRNA sequence are labeled in blue. The putative TRS is labeled in bold type, in purple and is underlined. Start codon AUG is labeled in red.

While the function of SARSr-CoV ORF7a protein remained to be elucidated, studies have shown that this accessory protein was expressed during SARS-CoV replication. ORF7a protein was shown to exit the endoplasmic reticulum through COPII transport machinery and is targeted to the Golgi apparatus [41]. To determine if ORF10 accessory gene is expressed in Rs-BatCoV HKU32, the leader-body junction sites and flanking sequences of ORF10 subgenomic mRNA were determined (Figure 5). The subgenomic mRNAs was successfully amplified and sequenced directly from both samples TLC26A and TLC28A. The sequences were aligned to the leader sequence, which confirmed 5’-CUAAAC-3’ as the core sequence of the TRS motif. The leader TRS and subgenomic mRNA of ORF10 accessory gene exactly matched each other.

## 4. Discussion

In this study, two novel alphaCoVs, Rs-BatCoV HKU32 from *Rhinolophus sinicus* in Hong Kong and Tr-BatCoV HKU33 from *Tylonycteris robustula* in Guizhou Province, were discovered. Their classification as novel species within the genus *AlphaCoV* is supported by the results from pairwise comparison of their 7 concatenated domains to those of known alphaCoVs based on the CoV species demarcation criteria by the ICTV. Phylogenetically, Rs-BatCoV HKU32 is most closely related to BtRf-AlphaCoV, BtMs-AlphaCoV, Ro-BatCoV HKU10 and Hi-BatCoV HKU10. On the other hand, Tr-BatCoV HKU33 is most closely related to BtNv-AlphaCoV, AlphaCoV BatCoV/P.kuhlii/Italy/206645-41/2011 and AlphaCoV BatCoV/P.kuhlii/Italy/206679-3/2010. Nevertheless, different phylogenetic positioning with closely related alphaCoVs was observed between ORF1ab and the S gene, suggesting that the S gene may have evolved in a path different from other genome regions. The S protein of CoVs is responsible for receptor recognition and contains epitopes for neutralizing antibodies, and hence is often subjected to selective pressure. Therefore, its evolutionary path may be different from that of other genome regions. For example, we have previously found that another bat alphaCoV, Rs-BatCoV HKU2, contains an evolutionarily distinct spike protein which is only distantly related to other alphaCoVs [33].

The close phylogenetic relationship between Rs-BatCoV HKU32 and BatCoV HKU10 may be explained by the geographical proximity of their hosts. Besides Rs-BatCoV HKU32 and Tr-BatCoV HKU33, diverse alphaCoVs and betaCoVs were also detected in our bat samples. Hi-BatCoV HKU10 and Coronavirus PREDICT CoV-37, belonging to alphaCoVs, were detected in bats in Hong Kong, while SARSr-BatCoVs and Ty-BatCoV HKU4, belonging to lineage B (*Sarbecovirus*) and C (*Merbecovirus*) betaCoVs, respectively, were detected in bats in Guangdong and Guizhou provinces. It is of note that Rs-BatCoV HKU32 and BatCoV HKU10 were detected in bats of different families but captured from the same sampling location at a country park in Hong Kong. This suggests that these viruses may have evolved among geographically close but phylogenetically distant bat populations. We have also previously described interspecies transmission of BatCoV HKU10 between *Rousettus leschenaultii* and *Hipposideros pomona* bats which belonged to two different bat families [42]. Given that the genetic cluster formed by Rs-BatCoV HKU32, BatCoV HKU10, BtRs-AlphaCoV and BtMs-AlphaCoV were detected from diverse bat families, this suggests that these alphaCoVs may have a tendency for cross-species transmission. This is in contrast to Tr-BatCoV HKU33 and related viruses which were limited to the family *Vespertilionidae*. Greater bamboo bats, the host of Tr-BatCoV HKU33, and other Vesper bats are geographically widespread and able to occupy distinct and diverse habitats [43]. Interestingly, MERS-CoV-related bat lineage C betaCoVs (*Merbecovirus*) have also been exclusively detected in the family *Vespertilionidae* [10,11,13,14,15,44,45]. Further studies are needed to understand the mechanism for host tropism, especially the stringency of host receptor usage by different alphaCoVs, the interspecies transmissibility of coronaviruses among different bats and the contribution of bat ecology to this phenomenon.

The presence of a homologous SARSr-CoV ORF7a-like protein in Rs-BatCoV HKU32 suggests a common evolutionary origin of this accessory protein from viruses in Chinese horseshoe bats (*Rhinolophus sinicus*). While they share the same host specificity for Chinese horseshoe bats, Rs-BatCoV HKU32 is an alphaCoV while SARSr-CoV is a lineage B betaCoV (*Sarbecovirus*). While the two proteins in SARSr-CoVs and Rs-BatCoV HKU32 share only 29% amino acid identity, they both possess typical conserved domains of a type I transmembrane protein. Moreover, this ORF10 (SARSr-CoV ORF7a-like) gene is shown to be expressed in Rs-BatCoV HKU32, suggesting it may play a role in viral replication. Besides SARSr-CoVs and Rs-BatCoV HKU32, another alphaCoV, Rs-BatCoV HKU2 has also been detected in this horseshoe bat species which is the origin of the SARS epidemic. Interestingly, Rs-BatCoV HKU2 was also recently found to have evolved and emerged in swine population causing epidemics in China, supporting Chinese horseshoe bats as an important animal source for CoV epidemics in both humans and animals [30,31,32]. This horseshoe bat species, which ranges from northern India to southern China, often resides in caves or any man-made cave-like structures such as abandoned tunnels in Hong Kong [43]. It is therefore important to preserve their natural habitats and avoid contact with these bats. Besides SARS-CoV and MERS-CoV, HCoV 229E, an alphaCoV, is also likely originated from bats approximately 219 to 333 years ago (Figure 4) [28], suggesting that alphaCoVs, like betaCoVs, are also the potential source for new epidemics in humans. Further studies are required to elucidate the function of SARSr-CoV ORF7a protein and the emergence potential of Rs-BatCoV HKU32.

## Figures and Tables

**Table 1:** Detection of CoVs in different bat species by reverse transcription (RT)-polymerase chain reaction (PCR) of the 440-bp fragment of RNA-dependent RNA polymerase (RdRp) gene.

Scientific Name	Common Name	No. of Bats Captured	No. of Bats Positive for CoV / (%)	CoV Detected	Sampling Location of Bats
*Cynopterus sphinx*	Greater short-nosed fruit bat	3	0	-	SWH
*Hipposideros armiger*	Great roundleaf bat	3	0	-	GZ
*Hipposideros larvatus*	Intermediate roundleaf bat	21	0	-	GZ
*Hipposideros pomona*	Pomona leaf-nosed bat	182	2 / (1.1)	Hi-BatCoV HKU10	TLC13, GD
*Hypsugo pulveratus*	Chinese pipistrelle	2	0	-	LMHP
*Miniopterus magnater*	Western bent-winged bat	1	0	-	SK01
*Miniopterus pusillus*	Small bent-wing bat	56	0	-	LMH, SWH, SK01
*Miniopterus schreibersii*	Common bent-wing bat	23	0	-	SK01
*Miniopterus filiginosus*	Eastern bent-wing bat	1	0	-	LMHP
*Myotis chinensis*	Large myotis	10	0	-	SK01, GZ
*Myotis ricketti*	Rickett’s big-footed bat	93	1 / (1.1)	Coronavirus PREDICT CoV-37	LMH01, SK01
*Nyctalus noctula*	Common noctule	1	0	-	YSO
*Pipistrellus abramus*	Japanese pipistrelle	6	0	-	MPO, YSO, KKSH
*Pipistrellus tenuis*	Least pipistrelle	4	0	-	KKSH, YSO, SWH, LMHP
*Rhinolophus affinis*	Intermediate horseshoe bat	76	0	-	TLC01, TLC13, SK01
*Rhinolophus pearsonii*	Pearson’s horseshoe bat	2	0	-	GDP
*Rhinolophus pusillus*	Least horseshoe bat	17	0	-	TLC13
*Rhinolophus sinicus*	Chinese horseshoe bat	272	10 / (3.7)	Rs-BatCoV HKU32 (7) SARSr BatCoV (3)	TLC01, GDP
*Tylonycteris pachypus*	Lesser bamboo bat	240	18 / (7.5)	Ty-BatCoV HKU4	WKT, PFL, SWH, TLC01, GZP
*Tylonycteris robustula*	Greater bamboo bat	104	1 / (0.96)	Tr-BatCoV HKU33	GZP

GD, Guangdong Province; GDP, Guangdong Province—Conghua City; GZ, Guizhou Province; GZP, Guizhou Province—Luodian County; KKSH, Kai Kuk Shue Ha, Luk Keng; LMH, Lin Ma Hang Lead Mine; LMHP, Lin Ma Hang Pool; MPO, Mai Po Nature Reserve; PFL, Pok Fu Lam; SK01, Sai Kung; SWH, Sheung Wo Hang, Sha Tau Kok; TLC01, Tai Lam-Shek Kong; TLC13, Tai Lam-Shek Kong; WKT, Wu Kau Tang; YSO, Yung Shue O Stream, Sai Kun.

**Table 2:** Coding potential and predicted domains in different proteins of Rs-BatCoV HKU32 strain TLC28A.

					Putative TRS	
ORF	Nucleotide Positions (Start–End)	No. of Nucleotides	No. of Amino Acids	Frame(s)	Nucleotide Position in Genome	TRS Sequence (Distance (No. of Bases) to AUG) ^1^
1ab	291–20,428	20,137	6712	+2, +3	69	AA**CUAAAC**(216)AUG
nsp1	291–875	585	195	+3		
nsp2	876–2963	2088	696	+3		
nsp3	2964–7649	4686	1562	+3		
nps4	7650–9083	1434	478	+3		
nsp5	9084–9989	906	302	+3		
nsp6	9990–10817	828	276	+3		
nsp7	10,818–11,066	249	83	+3		
nsp8	11,067–11,651	585	195	+3		
nsp9	11,652–11,975	324	108	+3		
nsp10	11,976–12,383	408	136	+3		
nsp11		51	17	+3		
nsp12	12,384–15,163	2780	927	+2		
nsp13	15,164–16,954	1791	597	+2		
nsp14	16,955–18,508	1554	518	+2		
nsp15	18,509–19,525	1017	339	+2		
nsp16	19,526–20,428	903	300	+2		
S	20,430–24,485	4056	1351	+2	20,421	AA**CUAAAU**(3)AUG
ORF3	24,485–25,153	669	222	+3	24,279	TC**CUUAAC**(199)**AUG**
ORF4	25,184–25,543	360	119	+2		
ORF5a	25,544–25,888	345	114	+2	25,540	GA**CUAAAU**G
ORF5b	25,782–26,225	444	147	+3		
E	26,209–26,433	225	74	+1	26,140	AA**CUAAAC**(64)AUG
M	26,440–27,126	687	228	+1	26,430	GT**CUAAAC**(4)AUG
N	27,137–28,279	1143	380	+2	27,128	AA**CUAAAC**(3)AUG
ORF9	28,251–28,568	318	105	+3	28,187	AG**CUGAAC**(58)AUG
ORF10 (SARS-CoV ORF7a-like protein)	28,593–28,955	363	120	+3	28,284	AA**CUAAAC**(303)AUG

^1^ TRS sequences are shown in bold.

**Table 3:** Characteristics of putative nonstructural proteins of open reading frame (ORF)1ab in Rs-BatCoV HKU32 strain TLC28A, Tr-BatCoV HKU33 strain GZ151867, BatCoV HKU10 and BtNv-AlphaCoV/SC2013.

		Amino Acids			
nsp	Putative Function or Domain	Rs-BatCoV HKU32 Strain TLC28A	Ro-BatCoV HKU10 183A	Tr-BatCoV HKU33 Strain GZ151867	BtNv-AlphaCoV/SC2013
nsp1	Unknown	M^1^ – A^195^	M^1^ – A ^195^	M^1^ – A^193^	M^1^ – A^193^
nsp2	Unknown	P^196^ – G^891^	K^196^ – G^888^	K^194^ – G^771^	K^194^ – G^771^
nsp3	ADRP, Putative PL^pro^ domains (PL1^pro^, PL2^pro^)	G^892^ – G^2453^	S^889^ – G^2518^	G^772^ – G^2339^	G^772^ – G^2338^
nsp4	Hydrophobic domain	S^2454^ – Q^2931^	S^2519^ – Q ^2996^	G^2340^ – Q^2817^	G^2339^ – Q^2815^
nsp5	3CL^pro^	S^2932^ – Q^3233^	S^2997^ – Q^3298^	S^2818^ – Q^3119^	A^2816^ – Q^3117^
nsp6	Hydrophobic domain	S^3234^ – Q^3509^	S^3299^ – Q^3574^	G^3120^ – Q^3398^	S^3118^ – Q^3395^
nsp7	Unknown	S^3510^ – Q^3592^	S^3575^ – Q^3657^	S^3399^ – Q^3481^	S^3396^ – Q^3478^
nsp8	Unknown	S^3593^ – Q^3787^	S^3658^ – Q^3852^	S^3482^ – Q^3676^	S^3479^ – Q^3673^
nsp9	Unknown	N^3788^ – Q^3895^	N^3853^ – Q^3960^	N^3677^ – Q^3784^	N^3674^ – Q^3781^
nsp10	Unknown	A^3896^ – Q^4031^	A^3961^ – Q^4097^	A^3785^ – Q^3919^	A^3782^ –Q^3916^
nsp11	Unknown	S^4032^ – D^4048^	A^4098^ – Q^4115^	T^3920^ – D^3936^	A^3917^ – D^3933^
nsp12	RdRp	S^4032^ – Q^4958^	A^4098^ – Q^5024^	T^3920^ – Q^4846^	A^3917^ – Q^4843^
nsp13	Hel	A^4959^ – Q^5555^	S^5025^ – Q^5621^	S^4847^ – Q^5443^	S^4844^ – Q^5440^
nsp14	ExoN, N7-MTase	S^5556^ – Q^6073^	A^5622^ – Q^6139^	A^5444^ – Q^5960^	S^5441^ – Q^5958^
nsp15	NendoU	G^6074^ – Q^6412^	S^6140^ – Q^6478^	S^5961^ – Q^6299^	G^5959^ – Q^6297^
nsp16	O-MT	A^6413^ – K^6712^	S^6479^ – R^6780^	S^6300^ – Y^6591^	S^6298^ – Y^6589^

**Table 4:** Cleavage site used between nsp in Rs-BatCoV HKU32 strain TLC28A, Tr-BatCoV HKU33 strain GZ151867, BatCoV HKU10 and BtNv-AlphaCoV/SC2013.

nsp	Cleavage Site			
	Rs-BatCoV HKU32 Strain TLC28A	Ro-BatCoV HKU10 183A	Tr-BatCoV HKU33 Strain GZ151867	BtNv-AlphaCoV/SC2013
nsp1/nsp2	A/P	A/K	A/K	A/K
nsp2/nsp3	G/G	G/S	G/G	G/G
nsp3/nsp4	G/S	G/S	G/G	G/G
nsp4/nsp5	Q/S	Q/S	Q/S	Q/A
nsp5/nsp6	Q/S	Q/S	Q/G	Q/S
nsp6/nsp7	Q/S	Q/S	Q/S	Q/S
nsp7/nsp8	Q/S	Q/S	Q/S	Q/S
nsp8/nsp9	Q/N	Q/N	Q/N	Q/N
nsp9/nsp10	Q/A	Q/A	Q/A	Q/A
nsp10/nsp12	Q/S	Q/A	Q/T	Q/A
nsp12/nsp13	Q/A	Q/S	Q/S	Q/S
nsp13/nsp14	Q/S	Q/A	Q/A	Q/S
nsp14/nsp15	Q/G	Q/S	Q/S	Q/G
nsp15/nsp16	Q/A	Q/S	Q/S	Q/S

**Table 5:** Pairwise comparison of *Coronaviridae*-wide conserved domains in replicase polyprotein 1ab and overall replicase polyprotein 1ab between Rs-BatCoV HKU32 strain TLC28A and other alphaCoVs.

Replicase Polyprotein Domain	Pairwise Sequence Identity with Rs-BatCoV HKU32 Strain TLC28A (%)				
	BtRf-AlphaCoV/ HuB2013	BtMs-AlphaCoV/ GS2013	Ro-BatCoV HKU10	PEDV	Tr-BatCoV HKU33 strain GZ151867
nsp3	67.6	67.6	60.3	50.1	49.0
nsp5	84.8	84.8	81.5	74.2	75.2
nsp12	92.6	92.6	90.1	83.2	83.7
nsp13	94.1	94.3	92.1	85.6	80.9
nsp14	93.4	93.4	90.0	79.9	78.8
nsp15	89.4	89.4	83.8	76.7	78.2
nsp16	89.7	90.0	85.8	82.8	81.2
7 Concatenated Domains	83.2	83.3	78.6	70.3	69.5
Overall replicase pp1ab	80.1	80.5	75.0	65.8	63.3

**Table 6:** Coding potential and predicted domains in different proteins of Tr-BatCoV HKU33 strain GZ151867.

					Putative TRS	
ORF	Nucleotide Positions (Start–End)	No. of Nucleotides	No. of Amino Acids	Frame(s)	Nucleotide Position in Genome	TRS Sequence (Distance (No. of Bases) to AUG) ^1^
1ab	278–20,052	19,774	6591	+1, +2	54	AA**CUAAAC**(218)AUG
nsp1	278–856	579	193	+2		
nsp2	857–2590	1734	578	+2		
nsp3	2591–7294	4704	1568	+2		
nps4	7295–8728	1434	478	+2		
nsp5	8729–9634	906	302	+2		
nsp6	9635–10,471	837	279	+2		
nsp7	10,472–10,532	249	83	+2		
nsp8	10,533–11,305	585	195	+2		
nsp9	11,306–11,629	324	108	+2		
nsp10	11,630–12,034	405	135	+2		
nsp11		51	17	+2		
nsp12	12,035–14,814	2780	927	+1		
nsp13	14,815–16,605	1791	597	+1		
nsp14	16,606–18,156	1551	517	+1		
nsp15	18,157–19,173	1017	339	+1		
nsp16	19,174–20,052	879	292	+1		
S	20,053–24,150	4098	1365	+1	20,049	GA**CUAAAU**G
ORF3	24,150–24,755	606	201	+3	23,876	AT**CUCAAC**(268)AUG
E	24,777–25,004	228	75	+3	24,763	TT**CUCAAC**(8)AUG
M	25,011–25,697	687	228	+3	25,001	GT**CUAAAC**(4)AUG
N	25,706–26,977	1272	423	+2	25,699	AA**CUAAAC**(1)AUG
ORF7	26,989–27,348	360	119	+1	26,982	AA**CUAAAU**(1)AUG

^1^ TRS sequences are shown in bold.

**Table 7:** Pairwise comparison of *Coronaviridae*-wide conserved domains in replicase polyprotein 1ab and overall replicase polyprotein 1ab between Tr-BatCoV HKU33 strain GZ151867 and other alphaCoVs.

Replicase Polyprotein Domain	Pairwise Amino Acid Sequence Identity with Tr-BatCoV HKU33 Strain GZ151867 (%)				
	BtNv-AlphaCoV/SC2013	BtRf-AlphaCoV/HuB2013	BtMs-AlphaCoV/GS2013	AlphaCoV BatCoV/P.kuhlii/Italy 206679-3/2010	Rs-BatCoV HKU32 Strain TLC28A
nsp3	58.8	48.7	48.8	57.3	49.0
nsp5	82.5	74.2	74.5	78.8	75.2
nsp12	86.3	83.5	83.6	86.4	83.7
nsp13	87.0	79.7	80.1	82.7	80.9
nsp14	84.0	79.3	79.3	82.4	78.8
nsp15	85.8	79.6	79.0	84.7	78.2
nsp16	87.3	80.8	80.5	84.2	81.2
7 Concatenated Domains	76.3	69.2	69.1	74.4	69.4
Overall replicase pp1ab	73.4	62.9	63.0	71.6	63.3

## Abbreviations

AlphaCoV	Alphacoronavirus
BetaCoV	Betacoronavirus
bp	Base-pair
CoVs	Coronaviruses
DeltaCoV	Deltacoronavirus
E	Envelope
GammaCoV	Gammacoronavirus
HCoV	Human coronavirus
Hi	Hipposideros
ICTV	International Committee on Taxonomy of Viruses
M	Membrane
MERS-CoV	Middle East Respiratory Syndrome coronavirus
N	Nucleocapsid
NCBI	National Center for Biotechnology Information
ORF	Open reading frame
PCR	Polymerase chain reaction
Pi	Pipistrellus
Pp	Polyprotein
RdRp	RNA-dependent RNA polymerase
Ro	Rousettus
RSK	Rhinolophus sinicus kidney
RSL	Rhinolophus sinicus lung
RT	Reverse transcription
S	Spike
SADS-CoV	Swine Acute Diarrhea Syndrome coronavirus
SARSr-CoV	Severe Acute Respiratory Syndrome related coronavirus
TRS	Transcription regulatory sequence
Ty	Tylonycteris
µL	Microliter

## References

[1] Lau S.K., Woo P.C., Li K.S., Huang Y., Tsoi H.W., Wong B.H., Wong S.S., Leung S.Y., Chan K.H., Yuen K.Y. (2005). Severe acute respiratory syndrome coronavirus-like virus in chinese horseshoe bats. Proc. Natl. Acad. Sci. USA.

[2] Zhao G.P. (2007). Sars molecular epidemiology: A Chinese fairy tale of controlling an emerging zoonotic disease in the genomics era. Philos. Trans. R Soc. Lond. B Biol. Sci..

[3] Reusken C.B., Haagmans B.L., Muller M.A., Gutierrez C., Godeke G.J., Meyer B., Muth D., Raj V.S., Vries L.S., Corman V.M. (2013). Middle east respiratory syndrome coronavirus neutralising serum antibodies in dromedary camels: A comparative serological study. Lancet Infect. Dis..

[4] Haagmans B.L., Al Dhahiry S.H., Reusken C.B., Raj V.S., Galiano M., Myers R., Godeke G.J., Jonges M., Farag E., Diab A. (2013). Middle east respiratory syndrome coronavirus in dromedary camels: An outbreak investigation. Lancet Infect. Dis..

[5] Chan J.F.W., Lau S.K.P., To K.K.W., Cheng V.C.C., Woo P.C.Y., Yuen K.-Y. (2015). Middle east respiratory syndrome coronavirus: Another zoonotic betacoronavirus causing sars-like disease. Clin. Microbiol. Rev..

[6] Lau S.K.P., Wong A.C.P., Lau T.C.K., Woo P.C.Y. (2017). Molecular evolution of mers coronavirus: Dromedaries as a recent intermediate host or long-time animal reservoir?. Int. J. Mol. Sci..

[7] Woo P.C., Wang M., Lau S.K., Xu H., Poon R.W., Guo R., Wong B.H., Gao K., Tsoi H.W., Huang Y. (2007). Comparative analysis of twelve genomes of three novel group 2C and group 2D coronaviruses reveals unique group and subgroup features. J. Virol..

[8] Woo P.C.Y., Lau S.K.P., Li K.S.M., Poon R.W.S., Wong B.H.L., Tsoi H.-w., Yip B.C.K., Huang Y., Chan K.-h., Yuen K.-y. (2006). Molecular diversity of coronaviruses in bats. Virology.

[9] Lau S.K., Li K.S., Tsang A.K., Lam C.S., Ahmed S., Chen H., Chan K.H., Woo P.C., Yuen K.Y. (2013). Genetic characterization of betacoronavirus lineage C viruses in bats reveals marked sequence divergence in the spike protein of pipistrellus bat coronavirus hku5 in Japanese pipistrelle: Implications for the origin of the novel middle east respiratory syndrome coronavirus. J. Virol..

[10] Woo P.C.Y., Lau S.K.P., Li K.S.M., Tsang A.K.L., Yuen K.-Y. (2012). Genetic relatedness of the novel human group c betacoronavirus to tylonycteris bat coronavirus hku4 and pipistrellus bat coronavirus hku5. Emerg. Micro. Infect..

[11] Corman V.M., Ithete N.L., Richards L.R., Schoeman M.C., Preiser W., Drosten C., Drexler J.F. (2014). Rooting the phylogenetic tree of middle east respiratory syndrome coronavirus by characterization of a conspecific virus from an african bat. J. Virol..

[12] Yang L., Wu Z., Ren X., Yang F., Zhang J., He G., Dong J., Sun L., Zhu Y., Zhang S. (2014). Mers-related betacoronavirus in vespertilio superans bats, China. Emerg. Infect. Dis..

[13] Anthony S.J., Gilardi K., Menachery V.D., Goldstein T., Ssebide B., Mbabazi R., Navarrete-Macias I., Liang E., Wells H., Hicks A. (2017). Further evidence for bats as the evolutionary source of middle east respiratory syndrome coronavirus. MBio.

[14] Lau S.K.P., Zhang L., Luk H.K.H., Xiong L., Peng X., Li K.S.M., He X., Zhao P.S., Fan R.Y.Y., Wong A.C.P. (2018). Receptor usage of a novel bat lineage c betacoronavirus reveals evolution of middle east respiratory syndrome-related coronavirus spike proteins for human dipeptidyl peptidase 4 binding. J. Infect. Dis..

[15] Luo C.M., Wang N., Yang X.L., Liu H.Z., Zhang W., Li B., Hu B., Peng C., Geng Q.B., Zhu G.J. (2018). Discovery of novel bat coronaviruses in south china that use the same receptor as middle east respiratory syndrome coronavirus. J. Virol..

[16] Van der Hoek L., Pyrc K., Jebbink M.F., Vermeulen-Oost W., Berkhout R.J.M., Wolthers K.C., Wertheim-van Dillen P.M.E., Kaandorp J., Spaargaren J., Berkhout B. (2004). Identification of a new human coronavirus. Nat. Med..

[17] Peiris J.S.M., Lai S.T., Poon L.L.M., Guan Y., Yam L.Y.C., Lim W., Nicholls J., Yee W.K.S., Yan W.W., Cheung M.T. (2003). Coronavirus as a possible cause of severe acute respiratory syndrome. Lancet.

[18] Woo P.C., Lau S.K., Chu C.M., Chan K.H., Tsoi H.W., Huang Y., Wong B.H., Poon R.W., Cai J.J., Luk W.K. (2005). Characterization and complete genome sequence of a novel coronavirus, coronavirus hku1, from patients with pneumonia. J. Virol..

[19] Woo P.C., Lau S.K., Lam C.S., Lau C.C., Tsang A.K., Lau J.H., Bai R., Teng J.L., Tsang C.C., Wang M. (2012). Discovery of seven novel mammalian and avian coronaviruses in the genus deltacoronavirus supports bat coronaviruses as the gene source of alphacoronavirus and betacoronavirus and avian coronaviruses as the gene source of gammacoronavirus and deltacoronavirus. J. Virol..

[20] Lau S.K.P., Woo P.C.Y., Li K.S.M., Tsang A.K.L., Fan R.Y.Y., Luk H.K.H., Cai J.-P., Chan K.-H., Zheng B.-J., Wang M. (2014). Discovery of a novel coronavirus, china rattus coronavirus hku24, from norway rats supports the murine origin of betacoronavirus 1 and has implications for the ancestor of betacoronavirus lineage A. J. Virol..

[21] Lai M.M.C., Cavanagh D. (1997). The molecular biology of coronaviruses. Advances in Virus Research.

[22] Brian D.A., Baric R.S. (2005). Coronavirus genome structure and replication. Current Topics in Microbiology and Immunology.

[23] De Groot R.J., Baker S.C., Baric R., Enjuanes L., Gorbalenya A.E., Holmes K.V., Perlman S., Poon L., Rottier P.J.M., Talbot P.J., King A.M.Q., Adams M.J., Carstens E.B., Lefkowitz E.J. (2011). Family Coronaviridae. Virus Taxonomy, Classification and Nomenclature of Viruses. Ninth Report of the International Committee on Taxonomy of Viruses.

[24] Zaki A.M., van Boheemen S., Bestebroer T.M., Osterhaus A.D., Fouchier R.A. (2012). Isolation of a novel coronavirus from a man with pneumonia in saudi arabia. N. Engl. J. Med..

[25] Van Boheemen S., de Graaf M., Lauber C., Bestebroer T.M., Raj V.S., Zaki A.M., Osterhaus A.D., Haagmans B.L., Gorbalenya A.E., Snijder E.J. (2012). Genomic characterization of a newly discovered coronavirus associated with acute respiratory distress syndrome in humans. MBio.

[26] Tao Y., Shi M., Chommanard C., Queen K., Zhang J., Markotter W., Kuzmin I.V., Holmes E.C., Tong S. (2017). Surveillance of bat coronaviruses in Kenya identifies relatives of human coronaviruses nl63 and 229e and their recombination history. J. Virol..

[27] Corman V.M., Baldwin H.J., Tateno A.F., Zerbinati R.M., Annan A., Owusu M., Nkrumah E.E., Maganga G.D., Oppong S., Adu-Sarkodie Y. (2015). Evidence for an ancestral association of human coronavirus 229e with bats. J. Virol..

[28] Pfefferle S., Oppong S., Drexler J.F., Gloza-Rausch F., Ipsen A., Seebens A., Muller M.A., Annan A., Vallo P., Adu-Sarkodie Y. (2009). Distant relatives of severe acute respiratory syndrome coronavirus and close relatives of human coronavirus 229e in bats, ghana. Emerg. Infect. Dis..

[29] Huang Y.W., Dickerman A.W., Pineyro P., Li L., Fang L., Kiehne R., Opriessnig T., Meng X.J. (2013). Origin, evolution, and genotyping of emergent porcine epidemic diarrhea virus strains in the United States. MBio.

[30] Zhou P., Fan H., Lan T., Yang X.L., Shi W.F., Zhang W., Zhu Y., Zhang Y.W., Xie Q.M., Mani S. (2018). Fatal swine acute diarrhoea syndrome caused by an hku2-related coronavirus of bat origin. Nature.

[31] Pan Y., Tian X., Qin P., Wang B., Zhao P., Yang Y.L., Wang L., Wang D., Song Y., Zhang X. (2017). Discovery of a novel swine enteric alphacoronavirus (seacov) in southern China. Vet. Microbiol..

[32] Gong L., Li J., Zhou Q., Xu Z., Chen L., Zhang Y., Xue C., Wen Z., Cao Y. (2017). A new bat-hku2-like coronavirus in swine, China, 2017. Emerg. Infect. Dis..

[33] Lau S.K., Woo P.C., Li K.S., Huang Y., Wang M., Lam C.S., Xu H., Guo R., Chan K.H., Zheng B.J. (2007). Complete genome sequence of bat coronavirus hku2 from chinese horseshoe bats revealed a much smaller spike gene with a different evolutionary lineage from the rest of the genome. Virology.

[34] Lau S.K.P., Wong E.Y.M., Tsang C.C., Ahmed S.S., Au-Yeung R.K.H., Yuen K.Y., Wernery U., Woo P.C.Y. (2018). Discovery and sequence analysis of four deltacoronaviruses from birds in the middle east reveal interspecies jumping with recombination as a potential mechanism for avian-to-avian and avian-to-mammalian transmission. J. Virol..

[35] Bankevich A., Nurk S., Antipov D., Gurevich A.A., Dvorkin M., Kulikov A.S., Lesin V.M., Nikolenko S.I., Pham S., Prjibelski A.D. (2012). Spades: A new genome assembly algorithm and its applications to single-cell sequencing. J. Comput. Biol..

[36] Altschul S.F., Gish W., Miller W., Myers E.W., Lipman D.J. (1990). Basic local alignment search tool. J. Mol. Biol..

[37] Lau S.K.P., Woo P.C.Y., Yip C.C.Y., Fan R.Y.Y., Huang Y., Wang M., Guo R., Lam C.S.F., Tsang A.K.L., Lai K.K.Y. (2012). Isolation and characterization of a novel betacoronavirus subgroup a coronavirus, rabbit coronavirus hku14, from domestic rabbits. J. Virol..

[38] Katoh K., Rozewicki J., Yamada K.D. (2017). Mafft online service: Multiple sequence alignment, interactive sequence choice and visualization. Brief. Bioinform.

[39] Guindon S., Dufayard J.F., Lefort V., Anisimova M., Hordijk W., Gascuel O. (2010). New algorithms and methods to estimate maximum-likelihood phylogenies: Assessing the performance of phyml 3.0. Syst. Biol..

[40] Nelson C.A., Pekosz A., Lee C.A., Diamond M.S., Fremont D.H. (2005). Structure and intracellular targeting of the sars-coronavirus ORF7A accessory protein. Structure.

[41] Pekosz A., Schaecher S.R., Diamond M.S., Fremont D.H., Sims A.C., Baric R.S. (2006). Structure, expression, and intracellular localization of the SARS-COV accessory proteins 7A and 7B. Adv. Exp. Med. Biol..

[42] Lau S.K., Li K.S., Tsang A.K., Shek C.T., Wang M., Choi G.K., Guo R., Wong B.H., Poon R.W., Lam C.S. (2012). Recent transmission of a novel alphacoronavirus, bat coronavirus hku10, from leschenault’s rousettes to pomona leaf-nosed bats: First evidence of interspecies transmission of coronavirus between bats of different suborders. J. Virol..

[43] Wong A.C.P., Li X., Lau S.K.P., Woo P.C.Y. (2019). Global epidemiology of bat coronaviruses. Viruses.

[44] Yang Y., Du L., Liu C., Wang L., Ma C., Tang J., Baric R.S., Jiang S., Li F. (2014). Receptor usage and cell entry of bat coronavirus hku4 provide insight into bat-to-human transmission of mers coronavirus. Proc. Natl. Acad. Sci. USA.

[45] Wang Q., Qi J., Yuan Y., Xuan Y., Han P., Wan Y., Ji W., Li Y., Wu Y., Wang J. (2014). Bat origins of MERS-COV supported by bat coronavirus hku4 usage of human receptor cd26. Cell. Host Microbe.

